# Improved Performance of Acoustically Actuated Magnetoelectric Antenna with FeGa/FeGaB Bilayer

**DOI:** 10.3390/mi15020190

**Published:** 2024-01-27

**Authors:** Kunqi Li, Qiaozhen Zhang, Yang Chang, Jian Wang, Huiling Liu, Songsong Zhang, Yuandong Gu

**Affiliations:** 1 School of Microelectronics, Shanghai University, Shanghai 200444, China; calamansi1998@shu.edu.cn (K.L.); jwwlw@shu.edu.cn (J.W.); liuhuiling@shu.edu.cn (H.L.); alex.gu@shu.edu.cn (Y.G.); 2 College of Information, Mechanical, and Electrical Engineering, Shanghai Normal University, Shanghai 200234, China; 1000484226@smail.shnu.edu.cn

**Keywords:** acoustic wave, finite element method, magnetoelectric antenna, magnetoelectric coefficient, multiphysics coupling

## Abstract

Acoustically actuated magnetoelectric (ME) antennas utilize acoustic wave resonance to complete the process of receiving and transmitting signals, which promotes the development of antenna miniaturization technology. This paper presents a bilayer magnetostrictive/AlN ME laminated antenna. The proposed laminated antenna uses the FeGa/FeGaB bilayer materials as magnetostrictive materials, which combine the advantages of soft magnetic properties of FeGa and the low loss of FeGaB. First, multiphysics modeling and analysis are performed for the proposed ME laminated antenna by finite element method (FEM). The positive/inverse ME effects and the influences of the volume ratio of the FeGa/FeGaB bilayer on the antenna performance are studied. The results show that the output voltage and ME coefficient of the FeGa/FeGaB bilayer magnetostrictive material with a volume ratio of 1:1 are 3.97 times and 195.8% higher than that of the single FeGaB layer, respectively. The eddy current loss is 52.08% lower than that of single-layer FeGa. According to the surface equivalence principle, the far-field radiation process is simulated. The results show that the gain of the ME antenna is 15 dB larger than that of the same-size micro-loop antenna, and the gain of the ME antenna is about −44.9 dB. The improved performance and magnetic tunability of the proposed bilayer magnetostrictive materials make ME antennas excellent candidates for portable devices and implantable medical devices.

## 1. Introduction

Antennas act as an omnipresent critical component in portable devices [1,2], such as smartphones and implantable medical devices [3]. One of the most challenging problems in the field of antenna design lies in antenna miniaturization [4,5,6]. Traditional antennas propagate electromagnetic (EM) waves from conductive currents and rely on EM wave resonance [7], which makes the antenna operating at an ultra-high frequency (UHF) typically larger than one-tenth of the wavelength of the EM wave (~102 mm) [1], which severely limits antenna miniaturization. Magnetoelectric (ME) antennas are a new type of miniaturized mechanical antenna, which operate at their acoustic resonance. Since the acoustic wavelength is five orders of magnitude shorter than the EM wavelength at the same operating frequency, the antenna operating at the acoustic resonance can be effectively miniaturized. Unlike conventional electrically small antennas that rely on current oscillation to radiate EM waves, the ME antennas combine piezoelectric and magnetostrictive effects to receive and transmit signals [8], which prevent the excessive storage of reactive energy between the antenna and the platform, thereby improving the radiation efficiency [7]. Recently, the low electrical loss and small size ME antennas have attracted wide attention.

Much research has studied ME composites with different structures and materials [9,10,11,12,13,14,15,16]. Lin et al. prepared the Terfenol-D/PZT ME composite structure; the results showed that when the DC bias field was 1200 Oe, the ME coefficient reached 6 V/cm/Oe [17]. Nevertheless, the Terfenol-D material with such a large magnetostrictive coefficient requires a bias field above 1000 Oe, which limits application in a weak magnetic field environment. Instead of the Terfenol-D, Bush et al. proposed a FeGa/PZT ME composite structure with an ME coefficient of 4.1 V/cm/Oe, corresponding to an optimal bias magnetic field of 250 Oe [18]. In order to obtain better microwave characteristics and reduce losses, Chen et al. studied the performance of the FeGaB/AlN structure, which showed a maximum ME coefficient of 2.81 V/cm/Oe when the DC bias field was 125 Oe [19]. The above previous studies only focus on a single magnetostrictive layer, which makes it difficult to achieve comprehensive performance optimization. Bilayer magnetostrictive materials combine the advantages of different magnetic materials, optimize performance, and obtain a tunable ME antenna. However, its design involves a complex layered structure and requires multiphysics coupling in terms of piezoelectric and magnetostrictive effects [20]. Thus, an accurate model and analysis of the ME antenna is necessary. Yao et al. proposed a multiphysics and multiscale model combining dynamics, electrodynamics, and magnetoelasticity to demonstrate the principle of the ME antenna [7,21]. Chen et al. simulated the antenna near-field information by finite element modeling (FEM) [22]. However, most of the reports focus on theoretical analysis and lack modeling analysis on far-field radiation.

Therefore, in this work, a bilayer magnetostrictive/AlN ME laminated antenna based on the strong ME coupling between EM and bulk acoustic waves (BAW) is proposed and investigated. The laminated antennas use the FeGaB/FeGa bilayer as magnetostrictive materials, which combine the soft magnetic properties of FeGa and the low loss of FeGaB [23,24,25]. A multiphysics FEM model for the proposed ME laminated antenna is built to analyze the positive/inverse ME effects, respectively. On this basis, the effect of the volume ratio of two magnetostrictive materials on the antenna performance is studied. The results indicate that when FeGa:FeGaB = 1:1, the ME coefficient of the antenna can be enhanced by 1.958 times, and the eddy current loss is 52.08% lower than that of single-layer FeGa. In addition, the near field around the ME antenna is simulated and the gain of the ME antenna is calculated. Because a magnetoelectric antenna is not a traditional electrical excitation model, it is not suitable for direct far-field analysis by traditional electromagnetic simulation software. According to the Huygens equivalence principle, the field generated by the radiant source outside the closed surface can be regarded as a superposition of the field generated by all the sub-wave sources on the closed surface. Therefore, a set of surface currents and magnetic flux densities around the antenna structure can be used as radiation sources to analyze the far-field radiation performance of ME antennas. The results demonstrate that the gain of the ME antenna is about 15 dB higher than that of the micro-loop antenna of the same size. Additionally, by adjusting the volume ratio of the two magnetostrictive materials, the gain of the ME antenna does not change significantly, and the gain is about −44.9 dB. The results suggest that using FeGaB/FeGa bilayer magnetostrictive materials can reduce the size of the antenna and improve the performance of the antenna. This makes laminated ME antennas have potential applications in portable devices, biomedical instruments [26], wireless communication systems, and so on.

## 2. Theoretic Background and Multiphysics Modeling

### 2.1. Magnetoelectric and Piezoelectric Constitutive Relations

Since the typical configuration of a ME antenna is a composite heterostructure composed of magnetostrictive and piezoelectric layers [8], both magnetostrictive and piezoelectric effects have to be considered. The relationship between stress and strain in the magnetostrictive layer is then described by the following equation:

(1) $$S=c_{H}:(\varepsilon_{el}-\varepsilon_{me})$$

where $c_{H}$, $\varepsilon_{el}$, and $\varepsilon_{me}$ are the stiffness matrix, elastic strain, and magnetostrictive strain. The relation between the strain of the magnetostrictive layer and its displacement gradient is

(2) $$\varepsilon =\frac{1}{2}[\nabla u+(\nabla u{)}^{T}]$$

The magnetostrictive strain $\varepsilon_{me}$ is expressed by isotropic quadratic function of magnetization field M. The formula is written as:

(3) $$\varepsilon_{me}=\frac{3\lambda_{s}}{2M_{s}^{2}}{dev(M⊗M)}_{ij}$$

where $\lambda_{s}$ is the saturation magnetostriction and $M_{s}$ is the saturation magnetization. Due to the strain of the magnetostrictive layer being nonlinearly related to the magnetic field and mechanical stress in the materials [27], in order to accurately describe the saturation trend of magnetostrictive material, the nonlinear magnetostrictive is introduced in this model. The nonlinear magnetization behavior in the magnetostrictive phase can be described by the following equations:

(4) $$M=M_{s}(|H_{eff}|)\frac{H_{eff}}{\left|H_{eff}\right|}L$$

(5) $$L=coth\left[\frac{3\chi_{m}|H_{eff}|}{M_{s}}\right]-\frac{M_{s}}{3\chi_{m}|H_{eff}|}$$

(6) $$H_{eff}=H+\frac{3\lambda_{s}}{\mu_{0}M_{s}^{2}}S_{el}M$$

(7) $$S_{el}=dev(c_{H}:\varepsilon_{el})$$

Equation (5) is the Langevin function. Where $\chi_{m}$ is the initial susceptibility, $H_{eff}$ is the effective magnetic field intensity including the effect of mechanical stress, $\mu_{0}$ is the permeability of the vacuum. Correspondingly, the total magnetic field intensity can be decomposed into two components, H and the mechanical stress contribution to the $H_{eff}$, where H is the applied magnetic field. The magnetization is related to the magnetic field by Maxwell’s equations.

(8) $$\left\{\begin{array}{c} B=\mu_{0}(H+M)\\ B=\nabla \times A\\ \nabla \times H=J_{e}+\sigma E+j\omega D\\ E=-j\omega A \end{array}\right.$$

where A is the magnetic vector potential, B is the magnetic flux density, H is the magnetic field strength, $J_{e}$ is the external current density, σ is the electrical conductivity and D is the electric displacement field.

As another component of a multiferroic composite, the piezoelectric layer completes the conversion between electrical energy and mechanical energy. The constitutive equation in the piezoelectric material is as follows:

(9) $$\left\{\begin{array}{c} \varepsilon =s_{E}S+d^{T}E\\ D=d_{E}S+\xi^{T}E \end{array}\right.$$

where $s_{E}$, $d_{E}$, and $\xi^{T}$ are vectors of the flexibility matrix, piezoelectric coefficient matrix, and permittivity, respectively. In order to simulate the physical properties of piezoelectric material, corresponding constraint conditions need to be used

(10) $$\left\{\begin{array}{l} \nabla \cdot D=\rho_{V}\\ E=-\nabla V \end{array}\right.$$

where $\rho_{V}$ is the free electric charge density, V is the electric potential.

### 2.2. Multiphysics Modeling of a ME Laminated Composite Antenna

**Figure 1:**
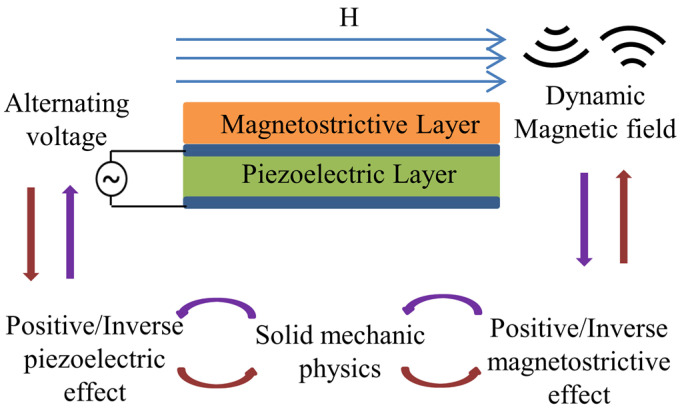
ME antenna working principle diagram. Alternating voltage is input/output through two layers of electrodes above and below the piezoelectric layer.

The fundamental elements of ME antennas include the piezoelectric layer and the magnetostrictive layer, as shown in Figure 1. In transmitting mode, an RF voltage is applied to the piezoelectric layer to stimulate an oscillating mechanical strain, which is transferred to the magnetostrictive layer to generate magnetization oscillation and radiate EM waves. Vice versa, in receiving mode, the magnetostrictive layer senses the dynamic magnetic field, generates an oscillating then transmits it to the piezoelectric layer, and finally realizes the output of RF voltage. On the basis of the positive/converse effects involved in the ME antenna, a multiphysics coupling modeling in terms of physics including electric field, magnetic field, and mechanical field is performed by commercial FEM software COMSOL 5.6.

**Figure 2:**
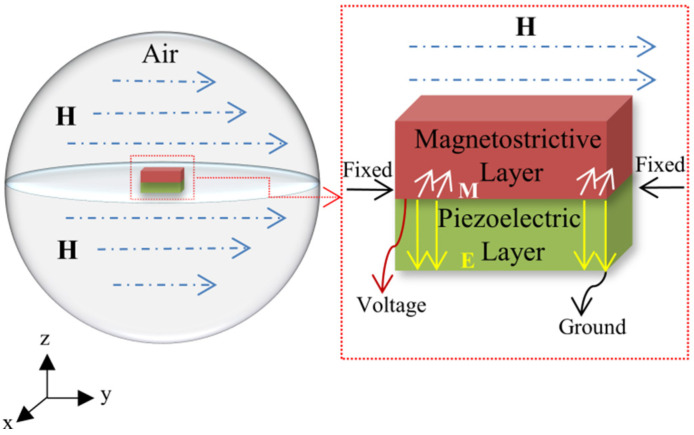
The FEM model and boundary conditions for ME antenna.

In order to simulate the working process of the ME antenna, it is necessary to solve two different kinds of multiphysics coupling, one is magnetostrictive physics, which combines solid mechanics with magnetic fields, and the other is piezoelectric physics, which combines solid mechanics with electrostatics. The calculation of multiphysics is based on the constitutive equations of the piezoelectric layer and magnetostrictive layer, respectively, coupled by adding appropriate boundary conditions in the solid mechanics, magnetic field, and electrostatic built-in modules of COMSOL. When studying the coupling of the antenna, only the effective region resonance of the functional layer is considered, and the lower electrode layer has little influence on the simulation results of magnetoelectric coupling. Therefore, in order to simplify the process, the simulation model mainly studies the piezoelectric layer and magnetostrictive layer. As shown in Figure 2, first, we establish an air domain and apply a DC magnetic field along the Y direction, which is because COMSOL cannot directly simulate the nonlinear inverse magnetostriction effect in the emission process. According to Equation (6), the effect of stress on magnetization requires that the magnetization intensity be specified first. This means that mechanical loads alone cannot magnetize the material, and a certain external magnetic field is required to calculate the inverse magnetostrictive effect. Then, fixed constraints are applied at both ends of the ME antenna and voltage is received and transmitted on both sides of the piezoelectric layer through voltage terminals and grounding.

**Figure 3:**
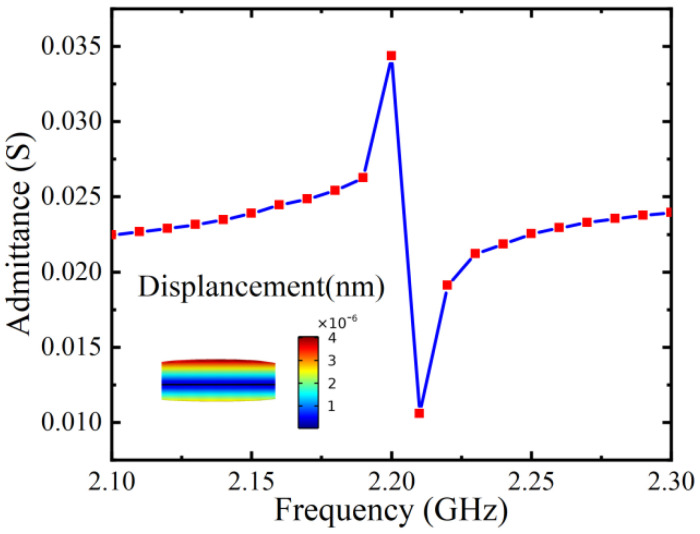
Admittance characteristic curve in thickness resonance mode (The illustration is a displacement cloud image under the thickness vibration mode).

Different resonant modes of BAW can be excited to coordinate the working frequency band of ME antennas. This paper studies the UHF ME antenna, which needs to excite the thickness resonance mode of BAW [1], as shown in Equation (11):

(11) $$f_{thickness}=\frac{1}{2t}\sqrt{\frac{E_{eq}}{\rho_{eq}}}$$

where t is the thickness of the piezoelectric layer, $E_{eq}$ is the equivalent Young’s modulus, and $\rho_{eq}$ is the equivalent density of the structure. When the magnetic field is applied in the Y direction, the ME antenna completes a voltage output due to the piezoelectric effect. Figure 3 shows the absolute admittance of the ME antenna and the mode pattern in the direction of thickness measured under the condition of AC/DC magnetic field superposition.

**Figure 4:**
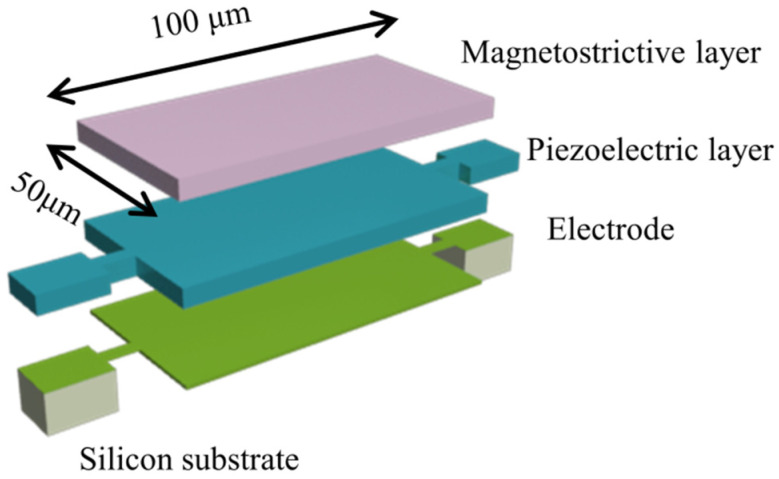
3D schematic of the ME antenna. Magnetostrictive material is used as the upper electrode.

The ME coefficient is one of the most important parameters to evaluate the efficiency of ME coupling [12,28].

(12) $$\alpha_{ME}=\frac{dV_{ME}}{t_{p}\times dH}$$

where $V_{ME}$ and $t_{p}$ are the voltage generated on the upper surface and the thickness of the piezoelectric layer, respectively. H is the AC and DC magnetic fields applied to the antenna. In previous studies, as a new magnetostrictive film material, the FeGa film, is widely used in ME devices due to its excellent magnetic softness and high conversion [29]. In this paper, a novel FeGaB/FeGa/AlN heterostructure is investigated. As shown in Figure 4, the antenna has planner dimensions of 100 μm × 50 μm, and the thicknesses of the AlN and FeGaB/FeGa layers are both 500 nm. Meanwhile, the total volume of magnetostrictive material is kept constant, and the volume ratio is changed to assess the role of using bilayer materials in ME devices. By combining FeGa thin film with FeGaB thin film, the overall microwave performance can be optimized to reduce eddy current losses on the premise of ensuring soft magnetic performance. Under the condition of a small magnetic field, it is expected to exhibit better ME conversion performance and high gain.

## 3. Results and Discussion

On the basis of multiphysics modeling, the performance in terms of receiving and transmitting process of ME antennas is simulated and analyzed in the following parts.

### 3.1. Positive ME Effect of the ME Antenna

When the ME antenna operates in receiving mode, the magnetostrictive layer senses the surrounding magnetic field, which stimulates magnetization oscillations, causing strain to be transferred to the piezoelectric layer, giving an alternating voltage output. To validate the simulation of the ME antenna, first, we use an example with the same size and material properties as developed in [19].

**Figure 5:**
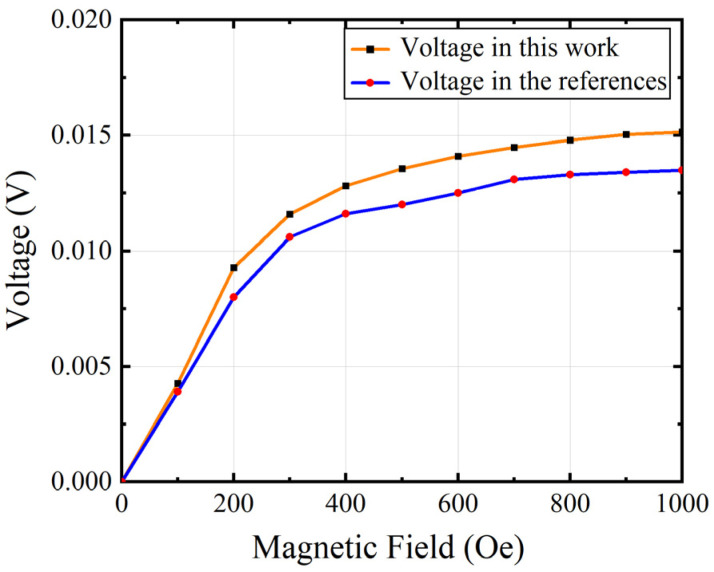
The comparison of the calculated output voltage with that of previously reported results in [19].

**Figure 6:**
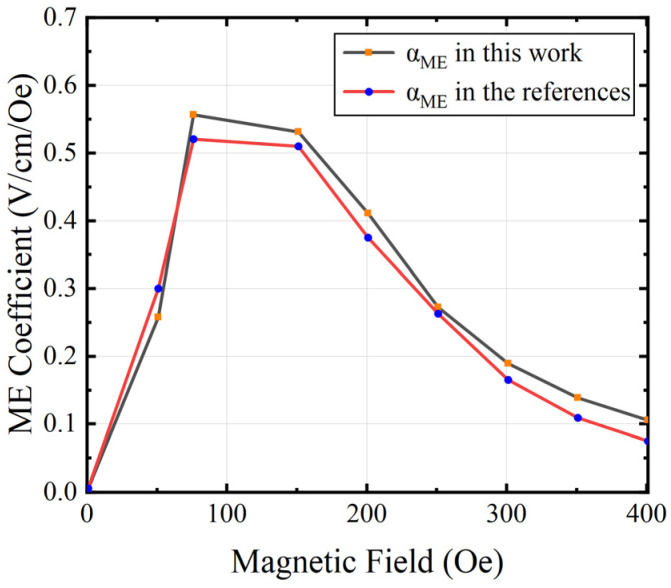
The comparison of the calculated ME coefficient ($\alpha_{ME}$) with that of previously reported results in [19].

The FEA model consists of an ME antenna with a device size of 100 μm × 50 μm, and the thickness of each layer is 1 μm. And DC magnetic field $H_{DC}$ is applied to the structure in the external air domain and $H_{DC}$ varies from 0 to 1000 Oe. The output voltage in [19] is compared with the simulated output voltage of our model in Figure 5. The results show that there is an acceptable slight deviation between the two curves, which may be due to the inconsistency of the mechanical loss and dielectric loss factors set in the simulation software. Figure 6 shows the ME coefficients of the model simulation in this paper and [19], respectively. The results show the same trend. When $H_{DC}$ is 75 Oe, the ME coefficient reaches the maximum, and the ME coefficient of our model reaches 0.55 V/cm/Oe, and in [19], the ME coefficient reaches 0.52 V/cm/Oe. The simulated results are in good agreement with those reported in [19].

After validation, the output voltage and ME coefficient of the proposed antenna using FeGa/FeGaB bilayer materials with different volume ratios *R* are studied. *R* is defined as:

(13) $$R=\frac{V_{FeGa}}{V_{FeGaB}}$$

**Figure 7:**
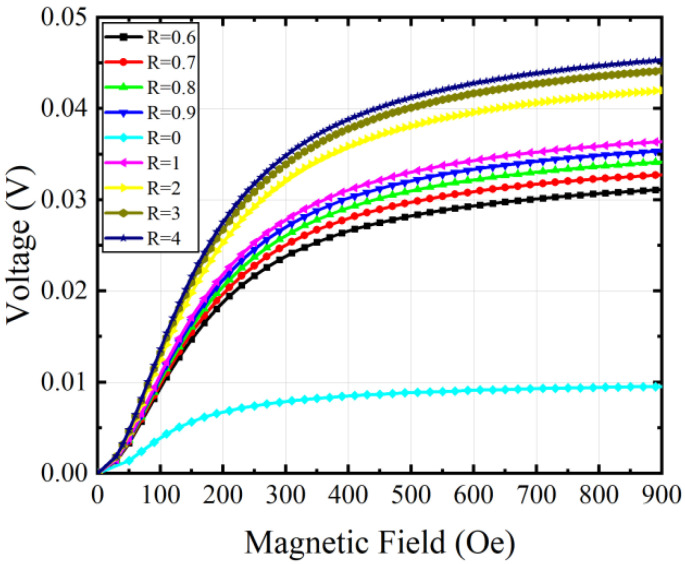
The output voltages of ME antennas with different volume ratios as $H_{DC}$ increases.

During the modeling, we applied a biased magnetic field in the Y direction to activate the L-T operating mode of the ME antenna, this field consists of a varying $H_{DC}$ and a $6\times 10^{-4}H_{AC}$ field. And its positive ME effect process is calculated by using the built-in stationary solver. Figure 7 shows the output voltage changes with different magnetic fields $H_{DC}$. As shown, the output voltage first increases and then approaches saturation with the increase in $H_{DC}$ due to the nonlinear magnetostrictive structure. It is of note that the output voltage of the ME antenna using the FeGa/FeGaB bilayer is much higher than that using the single-layer FeGaB material *(R* = 0). The output voltage is enhanced by 4.77 times when *R* changes from 0 to 4.

**Figure 8:**
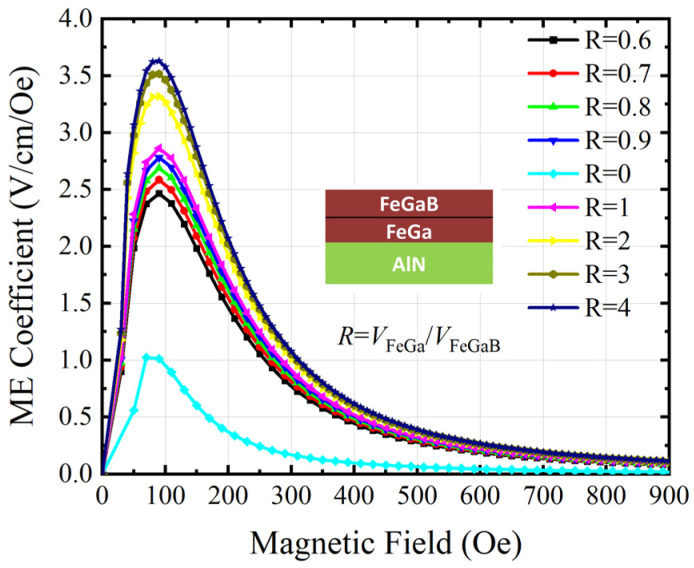
Simulated ME coefficient ($\alpha_{ME}$) of the ME antenna with different volume ratios under varying $H_{DC}$.

Figure 8 shows the calculated ME coefficient ($\alpha_{ME}$) of the ME antenna with different volume ratios under varying $H_{DC}$. It is seen that $\alpha_{ME}$ first increases to the maximum and then decreases with an increasing magnetic field. Among them, the $\alpha_{ME}$ changes from 1.014 V/(cm Oe) to 3.635 V/(cm Oe) when R is 4. Compared to the *R* = 0 case with the single layer FeGaB material, $\alpha_{ME}$ is enhanced by 285.5%. Moreover, the optimal bias magnetic field shifts to 90 Oe. This confirms that the ME conversion efficiency of ME antennas can be extremely improved by using FeGa/FeGaB laminated structures. Table 1 shows a performance comparison of the proposed state-of-the-art laminated ME antenna. It demonstrates that the laminated ME antenna exhibits a comparatively higher ME coefficient and smaller size.

**Figure 9:**
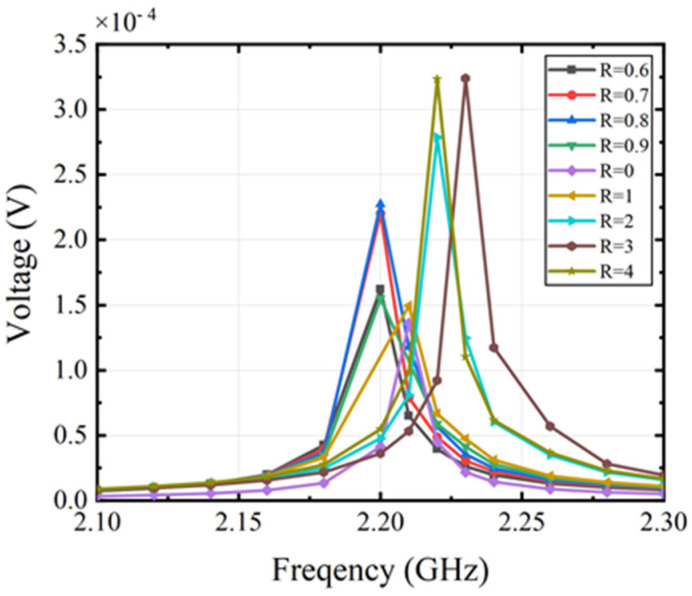
Simulated output voltage response of the ME antenna with different volume ratios.

In addition, we studied the output voltage response of the ME antenna at different frequencies. The simulation results illustrated in Figure 9 show the variation of the output voltage at different volume ratios. The results show that the output response is the strongest and the output voltage is the largest at the resonant frequency. The results verify that the resonant frequency of the device varies due to the different material properties and volume radios of the two materials. In addition, the output voltage amplitude is also improved.

**Figure 10:**
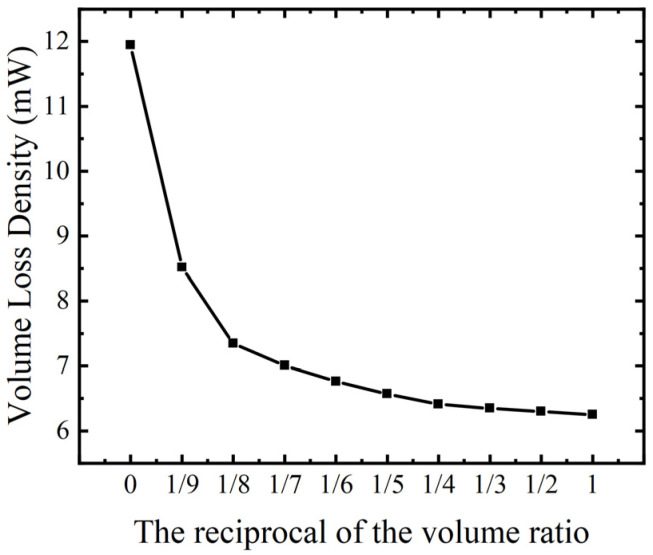
Eddy current loss curves corresponding to different volume ratios.

Because the laminated ME antenna combines the characteristics of the two materials, the good soft magnetic characteristics of FeGa and the relatively good microwave characteristics of FeGaB, the use of the FeGa/FeGaB structure not only improves the magnetoelectric performance compared with the use of single-layer FeGaB but also reduces the electrical loss generated by the use of single-layer FeGa material. Under the above conditions of AC/DC magnetic field superposition, we studied the eddy current loss under different volume ratios. As shown in Figure 10, when the volume ratio is 1, the decrease trend tends to be saturated, and the eddy current loss is reduced by 52.08%.

### 3.2. Inverse ME Effect of the ME Antenna

**Figure 11:**
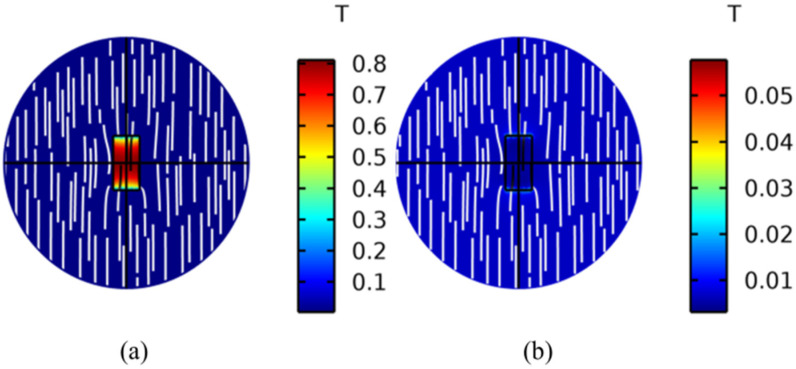
Simulation of flux density in (**a**) magnetostrictive layer and (**b**) piezoelectric layer. The maximum flux density of the magnetostrictive layer is distributed inside the material, and the maximum flux density of the piezoelectric layer is distributed outside the material.

In order to investigate the distribution of magnetic flux density in ME heterostructures, an RF voltage $V=V_{ac}\sin(\omega t)$ is applied to the piezoelectric layer. The RF voltage amplitude is 10 V and the frequency is 2.215 GHz. The results imply that the maximum flux density $B_{\max}$ of the magnetostrictive layer is distributed inside the material, and the $B_{\max}$ of the piezoelectric layer is distributed outside the material, as shown in Figure 11. This is owing to the magnetostrictive layer having a considerable magnetic permeability, which allows the concentration of higher flux density inside the material. However, the flux density at both ends of the piezoelectric layer is higher than that inside the material due to the external magnetic field.

**Figure 12:**
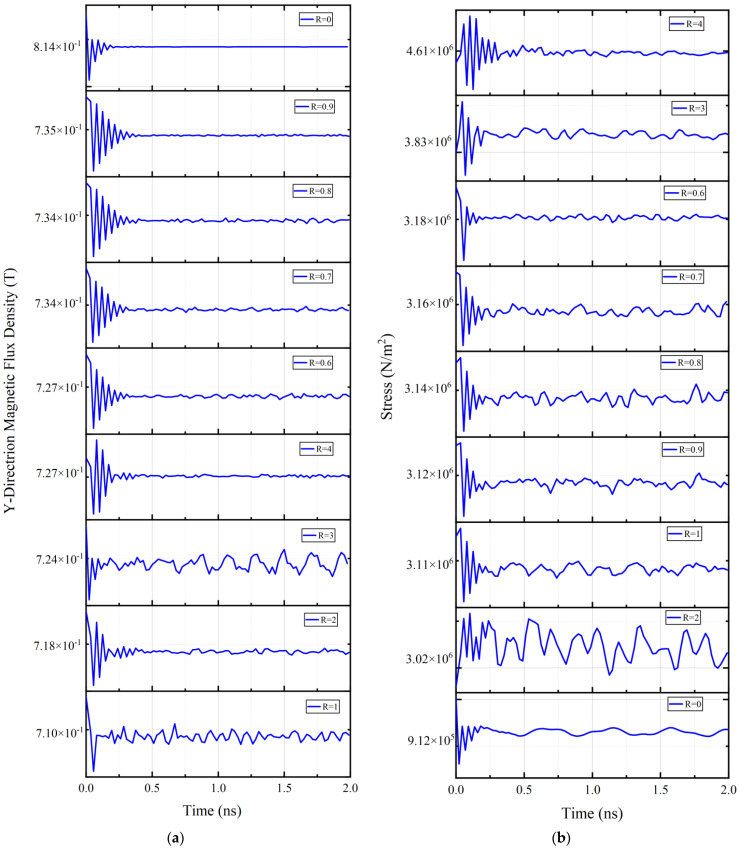
(**a**) Dynamic distribution of magnetostrictive layer flux density in Y direction; (**b**) Dynamic stress field of magnetostrictive layer.

Then the dynamic flux density distribution and stress distribution of the magnetostrictive layer are characterized. Under the condition that AC power is applied to the device, the transient calculation is carried out and the data values within 4 cycles are plotted and analyzed. Figure 12 shows the dynamic flux density changes and dynamic stress waveform of the magnetostrictive layer under different volume ratios, respectively. The results show that the flux density and the stress field amplitude decay with time as the AlN is electrically actuated. This indicates that the radiation of the electromagnetic wave acts as a damping load to the acoustic wave resonances.

The radiated power of the ME antenna is as follows:

(14) $$P_{\mathrm{rad}}=\frac{\omega^{2}t^{2}d_{31}^{2}}{2\eta }\iint_{s}{\left|T\right|}^{2}ds$$

where ω is the angular frequency, t is the thickness of the magnetostrictive layer, η is the wave impedance of the free space, $d_{31}$ is the piezomagnetic coefficient, T is the stress. According to the equation, it can be seen that increasing stress can improve the radiation efficiency of the ME antenna to a certain extent [30]. The laminated structure can improve the stress value at the interface to a certain extent, so the use of the laminated structure can improve the radiation efficiency of the antenna.

### 3.3. Far-Field of the ME Antenna

**Figure 13:**
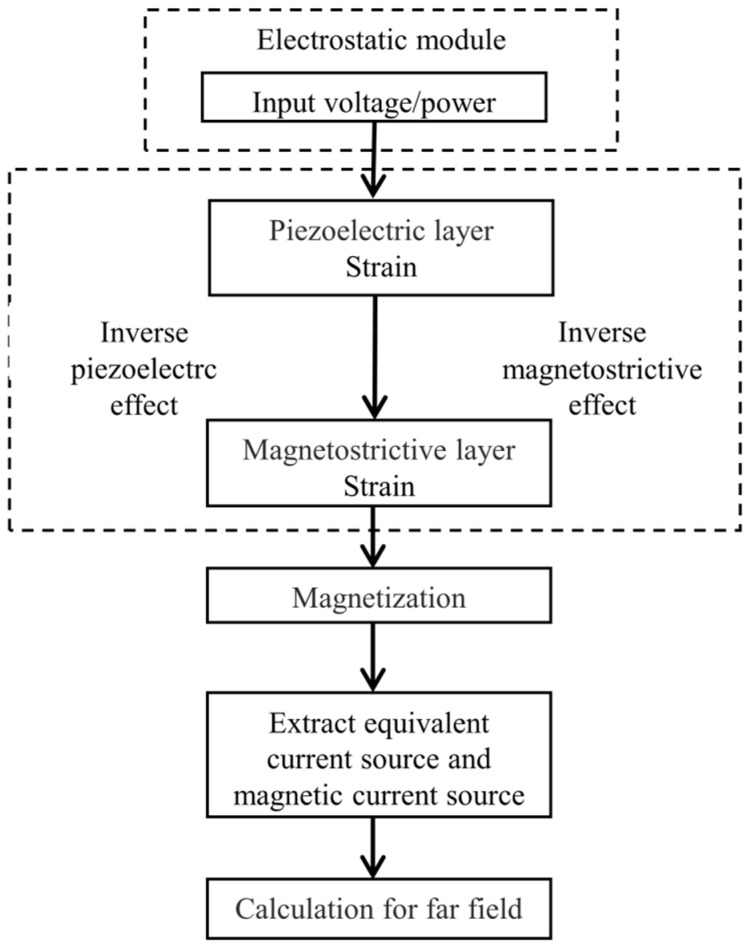
Flow of far-field calculation of ME antennas.

Gain is one of the key parameters to characterize the far-field performance of antennas. Since the ME antenna is not a traditional electric excitation source antenna, commercial simulation software cannot directly perform far-field calculations. In this work, we use the near-field equivalent source to calculate the far-field. Figure 13 shows the process of far-field simulation. To study the far-field radiation performance of the ME antenna, we first calculate the distribution of electric and magnetic fields in the near-field region of the antenna. According to the surface equivalence theorem, assuming that the materials and the surrounding environment are linear, a closed near-field region containing the ME antenna is taken as a Huygens box, and the near-field information of the surface is equivalent to a set of electric/magnetic current, which is used as the source of the Maxwell equation to calculate the gain of the antenna.

**Figure 14:**
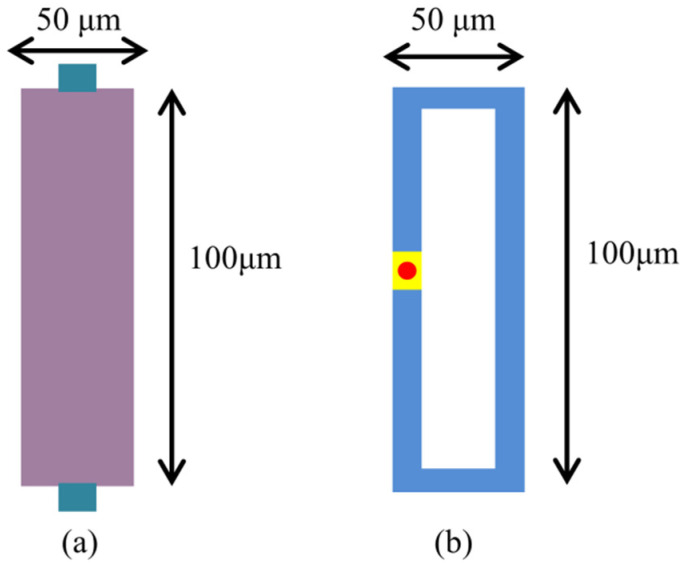
Two antennas with the same size 250 μm 50 μm, (**a**) ME antenna, (**b**) micro-loop antenna.

**Figure 15:**
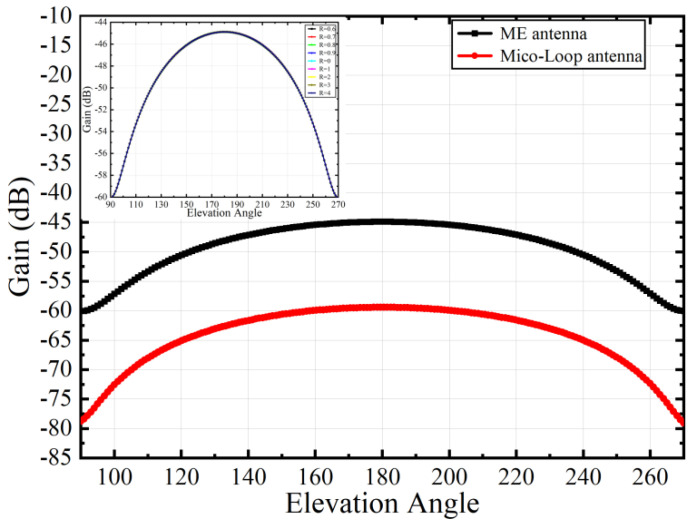
Comparison of gain between ME antenna and micro-loop antenna. Inset: Antenna gain of ME antennas with different volume ratios.

As a new type of miniaturized antenna, the far-field radiation performance of the ME antenna is better than that of the micro-loop antenna of the equivalent size. To verify this point, we construct a micro-loop antenna of the same size as the ME antenna, as shown in Figure 14, and apply electrical excitation to the port to calculate the gain. As shown in Figure 15, under the same excitation conditions, the maximum gain of the ME antenna using single-layer FeGaB material is −44.89 dB, while the maximum gain of the micro-loop antenna is −59.43 dB. The result shows that the gain of the ME antenna without lamination optimization is approximately 15 dB higher than that of the same-size micro-loop antenna.

Based on the above far-field calculation method, we calculate the gain of the layered ME antenna with different volume ratios. As the far-field calculation process requires coupling electrostatic, magnetic, solid mechanics, and RF modules, to simulate the radiation pattern of the ME antenna, an AC voltage with an amplitude of 1 V is applied to the upper side of the AlN layer, and background DC magnetic field $H_{DC}$ is set to 70 Oe. Prestress is calculated under steady conditions. The inverse ME process is calculated in the frequency domain. The electric/magnetic field on the Huygens surface is equivalent to the electric current $J_{s}$ and magnetic current $M_{s}$ through

(15) $$J_{s}=n\times H$$

(16) $$M_{s}=-n\times E$$

and eventually, the gain is calculated. The results show that the radiation pattern is similar to the magnetic-dipole antenna, the maximum occurs at 0° and 180°, and the minimum occurs at 90° and 270°. In order to simplify the process, a range of 90°–270°containing the maximum value is selected to plot the gain as a function of angle.

The inset in Figure 15 shows how the gain varies with the angle when the volume ratio of FeGa and FeGaB changes. The results demonstrate that the variation of antenna gain is almost unchanged when the volume ratio R is changed, and the curves almost overlap. When the volume ratio R becomes 1, the antenna gain reaches the maximum value of −44.88. The overall results show that the maximum gain of ME antennas using a laminated structure is higher than that of single-layer FeGaB. This is because the FeGaB/FeGa composite structure can reach higher inverse magnetoelectric conversion efficiency, thus obtaining a better far-field radiation performance.

## 4. Conclusions

In this paper, an acoustically actuated ME antenna based on FeGaB/FeGa bilayer magnetostrictive materials combined with AlN piezoelectric thin film is proposed, which improves both the ME coefficient and gain of the ME antenna. According to the coupling mechanism in the ME antenna, the multiphysics modeling and analysis of the proposed antenna is performed by FEM software COMSOL. Considering the comprehensive influence of the volume ratio of the layered structure on the performance of the ME antenna, better magnetoelectric conversion efficiency can be obtained when *R* = 1, the magnetoelectric coefficient of the single-layer FeGaB structure is increased by 1.958 times, the eddy current loss is reduced by 52.08% compared with the single-layer FeGa structure, and the far-field radiation performance is also optimized at this time. This technique also validates that the bilayer materials structure can increase the stress, which can enhance the average radiated power. The far-field simulation results show that the radiation pattern of the ME antenna is similar to that of a magnetic dipole. Meanwhile, the gain is improved by using FeGaB/FeGa bilayer magnetostrictive materials. These investigation results provide insights into the design of miniature tunable ME antennas with improved performance and promote the feasibility of ME antennas in portable devices, biomedicine, and wireless communications. Additionally, the proposed multiphysics FEM model can also provide the possibility of the design of a high-performance ME antenna with arbitrary structures and materials.

## Figures and Tables

**Table 1:** Comparison of structure and performance.

Work	Area (mm^2^)	Structure	Frequency	*α*_ME_ (V/cm/Oe)
Mukherjee	17.5	Metglas/PVDF/Metglas	49.9 kHz	0.1335
Viswan	9 × 10^−6^	Metglas/ZnO	1 kHz	0.047
Zheng	NA	NiFe_2_O_4_/Pb(Zr_0.52_Ti_0.48_) O_3_	10 MHz	0.24
Jian	NA	PbZr_0_._52_Ti_0.48_O_3_/NFO/PZT	100 kHz	1.2
Dong	84	Fe_80_Ga_20_/PMN-PT	1 kHz	1.01
Finkel	16	Fe_81.4_Ga_18.6_/PIN-PMN-PT	1 Hz	1.38
Chen	0.005	FeGaB/AlN	2.2 GHz	2.81
This work	0.005	Fe_7_Ga_2_B_1_/Fe_81_Ga_19_/AlN	2.215 GHz	3.835

## Data Availability

The data that support the findings of this study are available from the corresponding author upon reasonable request.
